# Comparison of the Multiattribute Utility Instruments EQ-5D and SF-6D in a Europe-Wide Population-Based Cohort of Patients with Inflammatory Bowel Disease 10 Years after Diagnosis

**DOI:** 10.1155/2016/5023973

**Published:** 2016-08-18

**Authors:** Gert Huppertz-Hauss, Eline Aas, Marte Lie Høivik, Ebbe Langholz, Selwyn Odes, Milada Småstuen, Reinhold Stockbrugger, Geir Hoff, Bjørn Moum, Tomm Bernklev

**Affiliations:** ^1^Department of Gastroenterology, Telemark Hospital, 3710 Skien, Norway; ^2^Department of Health Management and Health Economics, Institute of Health and Society, University of Oslo, 0310 Oslo, Norway; ^3^Health Services Research Unit, Akershus University Hospital, Lørenskog, Norway; ^4^Department of Gastroenterology, Oslo University Hospital, Ullevål, 0424 Oslo, Norway; ^5^Department of Medicine, Gentofte Hospital, 2900 Hellerup, Denmark; ^6^Ben-Gurion University of the Negev, 84105 Beersheba, Israel; ^7^Department of Biostatistics, Oslo University Hospital, 0424 Oslo, Norway; ^8^Department of Internal Medicine, University Hospital of Ferrara, 44124 Ferrara, Italy; ^9^Department of Research and Development, Telemark Hospital, 3710 Skien, Norway

## Abstract

*Background*. The treatment of chronic inflammatory bowel disease (IBD) is costly, and limited resources call for analyses of the cost effectiveness of therapeutic interventions. The present study evaluated the equivalency of the Short Form 6D (SF-6D) and the Euro QoL (EQ-5D), two preference-based HRQoL instruments that are broadly used in cost-effectiveness analyses, in an unselected IBD patient population.* Methods*. IBD patients from seven European countries were invited to a follow-up visit ten years after their initial diagnosis. Clinical and demographic data were assessed, and the Short Form 36 (SF-36) was employed. Utility scores were obtained by calculating the SF-6D index values from the SF-36 data for comparison with the scores obtained with the EQ-5D questionnaire.* Results*. The SF-6D and EQ-5D provided good sensitivities for detecting disease activity-dependent utility differences. However, the single-measure intraclass correlation coefficient was 0.58, and the Bland-Altman plot indicated numerous values beyond the limits of agreement.* Conclusions*. There was poor agreement between the measures retrieved from the EQ-5D and the SF-6D utility instruments. Although both instruments may provide good sensitivity for the detection of disease activity-dependent utility differences, the instruments cannot be used interchangeably. Cost-utility analyses performed with only one utility instrument must be interpreted with caution.

## 1. Introduction

Crohn's disease (CD) and ulcerative colitis (UC) are noninfectious chronic inflammatory bowel diseases (IBDs). UC affects the large intestine, whereas CD can affect all parts of the gastrointestinal tract. The typical symptoms of IBDs are diarrhoea, bloody stool, abdominal pain, urgency, fever, and weight loss [1]. Extraintestinal manifestations are possible, which most often affect the joints, skin, eyes, liver, or bile ducts [2]. Complicated CD is characterised by fistulae, abscesses, and stenosis, and 3–17% of UC patients [3] and 30–50% of CD patients undergo surgery within the first ten years after diagnosis [4]. Although recent studies have suggested that UC patients have decreased risks of colorectal cancer (CRC), the overall annual CRC risk still ranges between 0.06 and 0.16%, with a relative risk of 1.05 to 2.75 compared with that of the general population [5].

Because IBD is a lifelong disease, the aims of today's therapy are not only symptomatic improvement with better health-related quality of life (HRQoL) but also mucosal healing and a reduction in both the need for surgery and risk for CRC [6]. Hospitalisation and surgery contribute considerably to the direct costs of IBD treatment [7], and the cost effectiveness of expensive long-term medical treatment is poorly documented [8].

Limited resources are used as an argument for the mandatory implementation of cost-effectiveness analyses (CEAs) to prioritise different health programs [9]. Preference-based measures of health, or multiattribute utility measures (MAUs), are multidimensional classification systems of self-reported general health along with predefined weights for preference or utility. MAUs are broadly used to evaluate the health effects of therapeutic interventions by calculating Quality Adjusted Life Year (QALY) gains [10, 11]. Several MAU instruments exist, but they exhibit considerable variation in the dimensions covered [11–13], resulting in controversy as to which instruments might be more suitable for evaluating utility [9, 10]. The Short Form 6D (SF-6D), which was derived from the Short Form Health Survey 36 (SF-36), and the Euro QoL (EQ-5D) are frequently used utility instruments [14]. Evaluations have indicated poor sensitivity to changes of utility in the low range of values using the SF-6D (“floor effect”) [14–16]. Similarly, the EQ-5D form has low sensitivity for detecting changes in the upper range of values (“ceiling effect”) [14–16]. Utility assessments with the EQ-5D provide higher score values than the SF-6D in some studies [17, 18] but lower values in others [14, 19–21]. Moreover, changes occurring after interventions that are assessed with the EQ-5D may be larger than those assessed with the SF-6D [16, 19–21]. A possible consequence of this observation could be that the results of cost-utility analyses depend on the utility instrument chosen [19, 21]. However, the SF-6D might be more sensitive at detecting small utility changes compared to the EQ-5D, particularly in conditions with relatively good HRQoL [17, 22]. HRQoL has been reported to be rather good in population-based studies with IBD cohorts [23–25]. Thus, an instrument with good sensitivity for changes in the upper range of HRQoL scores seems to be important for assessing utility in population-based IBD cohorts.

The aim of the present study was to cross-sectionally assess the differences of the SF-6D and EQ-5D utility measures in an unselected IBD patient cohort in terms of both their descriptive systems and scoring distributions. Furthermore, we wanted to explore the differences between the EQ-5D and SF-6D in their ability to capture HRQoL score variations depending on clinical activity measures. The hypothesis was that the SF-6D is more sensitive than the EQ-5D to differences in the HRQoL according to clinical factors.

## 2. Patients and Methods

### 2.1. Patients

The present study was a part of the European Collaborative Study Group on Inflammatory Bowel Disease (EC-IBD) [26]. From October 1991 to September 1993, 2201 patients with newly diagnosed IBD from 20 well-defined areas in 12 European countries and Israel were included in a population-based, prospective, and uniformly diagnosed inception cohort. IBD was determined using the diagnostic criteria of Lennard-Jones [1].

The data for the 10-year follow-up were collected from August 2002 to January 2004. From the original 20 centres, only thirteen centres from nine countries contributed data from 1580 IBD patients. To reduce the possibility of selection bias, a minimum response rate of 60% was defined for each centre; this threshold was met by nine centres from seven countries (Oslo, Norway; Copenhagen, Denmark; Maastricht, the Netherlands; Vigo, Spain; Cremona and Reggio Emilia, Italy; Ioannina and Heraklion, Greece; and Beersheba, Israel).

### 2.2. Methods

All patients included in the study were invited to a standardised 10-year follow-up visit between August 2002 and January 2004 at their respective hospitals. At the visit, clinical and demographic data were obtained. Additional investigations, such as colonoscopies, were performed if necessary. All patients completed a questionnaire that included the SF-36 and EQ-5D forms. SF-6D scores were calculated based on the SF-36 scores [27]. Patient data were recorded by the patients through a web-based form, which had been previously presented and explained to them at the clinic [28]. Disease activity was registered as the presence or absence of current symptoms and the number of flares in the previous year, which were then dichotomised into flares or no flares in the previous year. Additionally, the level of subjectively perceived general health was derived from question one of the SF-36: “In general, would you say your health is: excellent, very good, good, fair, poor?”

### 2.3. Instruments

The EQ-5D contains five domains (mobility, self-care, usual activities, pain/discomfort, and anxiety/depression), with three levels in each domain describing no problems, some problems, or severe problems. Thus, 243 different health states can be described. Valuation was performed with the time-trade-off (TTO) method with UK tariffs. Utility scores were computed with the MVH-A1 algorithm [29], and the possible scores ranged from −0.59 to 1. Eleven items of the SF-36 version one were used to derive the SF-6D scores in six dimensions (physical functioning, role limitation, social functioning, pain, mental health, and vitality). Each dimension contained four to six levels describing the different degrees of impaired functioning. Thus, 18,000 different health states could be described. The standard gamble (SG) method was used in the valuation process for the SF-6D. Utility scores were computed using UK tariffs provided by Brazier and colleagues [30], with possible scores ranging from 0.29 to 1. Both instruments define a utility index of one as full health status, whereas zero is equivalent to death. The EQ-5D allows for scores smaller than zero, indicating the existence of health states worse than death.

### 2.4. Statistical Analysis

Because of the skewed distribution of the score values (particularly for the EQ-5D), we tested for correlations between the SF-6D and EQ-5D and their domains using nonparametric Spearman's rho tests. We examined the distributions of the health states of the entire sample using both instruments across all dimensions. Additionally, in patients classified as being in “full health” with one instrument, the distribution of health states obtained with the other instrument was assessed. Preference-based index values for each instrument are presented as the mean and the median with ranges. Agreement between the SF-6D and EQ-5D scores was assessed with the single-measure intraclass correlation coefficient (ICC, two-way random effects model, absolute agreement) and the Bland-Altman plot [31, 32]. Pairwise comparisons of score values that depended on disease activity were performed with *t*-tests, ANOVAs, or Mann-Whitney-Wilcoxon tests, as appropriate. The ability of the two instruments to detect clinically relevant utility differences was studied with Norman's criteria of clinical relevance, which demands a difference between two means to be larger than half the standard deviation [33], and with receiver operating characteristic (ROC) curves [34]. The area under the ROC curves (AUCs) described the discriminative properties of the instruments. The ideal discriminative ability is reached with an area of 1.0, while an area < 0.5 suggests no discriminative ability of an instrument beyond random chance. The sample size required to detect a statistically significant difference between utility index scores when comparing two subjectively perceived health state levels with a power of 0.90 and a significance level of 0.05 was estimated to be 85 in each group. Correction for multiple testing was performed using the Fisher's least significant difference (LSD) method. *P* values < 0.05 were considered to be statistically significant. All statistical tests were two-sided. Statistical analyses were performed using SPSS version 19 (IBM SPSS Statistics, Chicago, IL, USA) for Windows.

The funding sources of the study had no influence on the concept or implementation of the study.

## 3. Results

At the 10-year follow-up, nine centres from seven countries contributed data from 1199 patients. Of these, 115 (9.6%) patients were deceased, and eight patients had their IBD diagnosis withdrawn or had uncertain diagnoses, leaving 1076 eligible patients for inclusion. However, 307 patients did not complete the questionnaires because they were unwilling to participate or were untraceable. Thus, 769 IBD patients (71.5%), approximately of which two-thirds had UC (*n* = 517) and one-third had CD (*n* = 252), completed the questionnaires and were eligible for analysis. No differences in gender, diagnosis, age, disease distribution in the gastrointestinal tract, or disease complications (fistulising or stricturing CD) between responders and nonresponders were observed (data not shown). Utility scores measured with both utility instruments were significantly lower for females than for males and for CD patients than for UC patients (data not shown).

### 3.1. Dimension-to-Dimension Comparison

The correlations between EQ-5D and SF-6D dimension scores varied from 0.10 to 0.66 (Table 1, *P* < 0.01). Role limitation/usual activities and pain/pain and discomfort exhibited the highest correlations, whereas mental health/self-care and vitality/self-care exhibited the lowest.

The distributions of dimension scores of both utility instruments, particularly of EQ-5D, were skewed toward higher scores, indicating a relatively good HRQoL (Table 2). The majority of the dimensional health states in the EQ-5D were registered in level one and a minority of states were registered in levels two and three. The SF-6D dimensional scores were spread over three to four health state levels. Using the EQ-5D, 321 patients scored in the best possible condition (EQ-5D index score 1.0), whereas only 30 exhibited full health according to the SF-6D. In the SF-6D, 24% to 80.4% of patients with the best possible EQ-5D score were estimated to not be in perfect health according to dimensions of physical functioning, pain, mental health, and vitality (Table 3). Twenty-nine of the 30 patients who had the best possible SF-6D score also had the highest possible EQ-5D score.

### 3.2. Preference-Based Index Comparison

**Figure 1:**
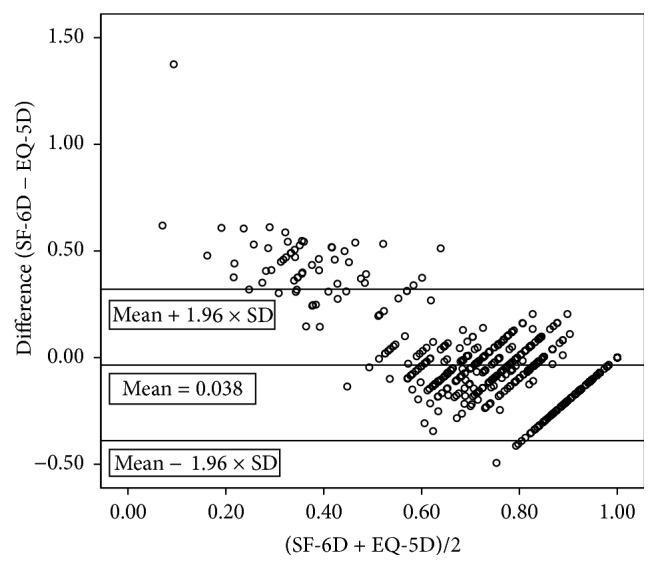
Bland-Altman plot of the differences in utility scores between SF-6D and EQ-5D.

The mean EQ-5D index score for all patients was 0.81 with a range of 1.59 (−0.59 to 1). The median EQ-5D score was 0.85. The mean SF-6D index score for all patients was 0.77 with a range of 0.62 (0.38 to 1). The median SF-6D index score was 0.79. Spearman's rho correlation coefficient between the EQ-5D and SF-6D index scores was 0.68 (*P* < 0.001). The Bland-Altman plot (Figure 1) displayed a nonrandom mean difference between the SF-6D and EQ-5D scores of −0.035, with a considerable number of values beyond the limits of agreement (mean ± 2SD = −0.035 ± 0.36). The difference increased with decreasing mean scores, indicating decreasing HRQoL. The single-measure ICC was 0.58, indicating that 42% of the total variability of results represented within-subject variability, that is, the variability between the two different utility instruments [31].

### 3.3. Disease Activity

Patients with self-reported IBD symptoms or flares in the previous year had statistically significantly lower median and mean SF-6D and EQ-5D index scores than those without (Table 4). However, the median and mean differences between these groups calculated from the EQ-5D scores were higher than those calculated from the SF-6D scores. The mean EQ-5D scores and SF-6D scores stratified by countries were significantly higher with symptoms than without (Table 5). In most of the countries flares in the previous year did not lead to significantly lower EQ-5D or SF-6D scores (Table 5). No difference was observed between the AUCs calculated for both MAU instruments, indicating that neither is superior at detecting utility differences that depend on current symptoms or flares. However, considerable differences were observed between the median and mean EQ-5D score values, particularly in patients without current symptoms (median 1.0; mean 0.88; Table 4).

**Figure 2:**
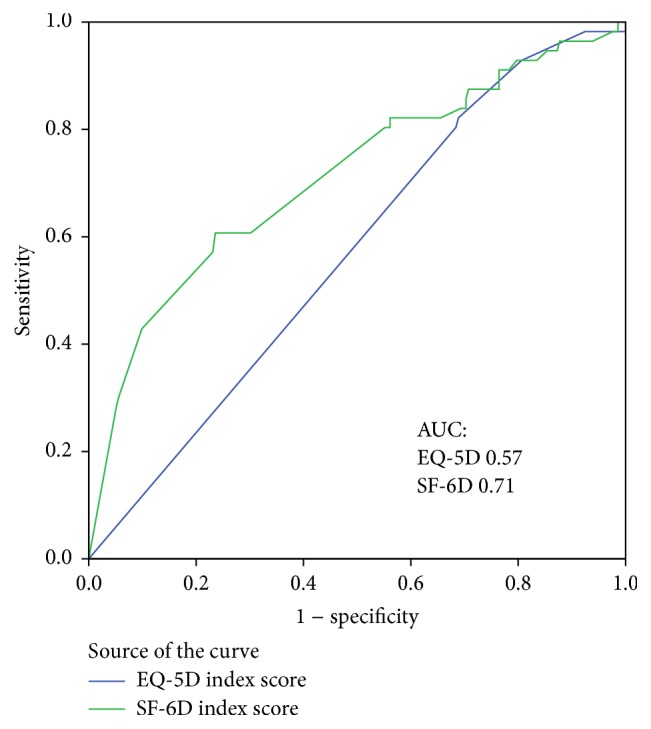
Receiver operating characteristic (ROC) curves of SF-6D and EQ-5D. AUC: area under the ROC curves of SF-6D and EQ-5D index scores that depend on self-perceived general health. The discriminative ability of SF-6D and EQ-5D between patients in excellent health and patients in very good health.

The SF-6D and EQ-5D were both able to detect statistically significant utility differences depending on subjectively perceived health derived from question one in the SF-36 (Table 6). However, in contrast to the SF-6D, the EQ-5D was unable to detect a statistically significant difference in the index scores between patients in “excellent” health and patients in “very good” health, with identical median score values of one and mean score values of 0.96 and 0.93, respectively. Additionally, in the same patient groups the AUC for the SF-6D using “very good health” as a cut-off point was larger than that for the EQ-5D (0.71, 95% CI 0.63 to 0.79 versus 0.57, 95% CI 0.49 to 0.65; Figure 2). This result indicated that the ability of the SF-6D to discriminate between “excellent” and “very good” health conditions in IBD patients was better than that of the EQ-5D. However, due to an overlap of the confidence intervals, the clinical significance of these results remains uncertain.

## 4. Discussion

In this European population-based IBD cohort, the EQ-5D and SF-6D utility instruments were both able to detect utility differences that depend on self-perceived disease activity and self-estimated general health. However, the results indicated a low degree of concordance between the two instruments.

Brazier et al. argued for a potentially high correlation between the SF-6D and EQ-5D dimensions with similar contents, such as physical functioning and mobility or pain and pain/discomfort (Table 1, correlation coefficients in bold) [14]. However, their study in seven cohorts with different diseases and van Stel's study in a coronary heart disease cohort were only able to register poor to moderate correlation coefficients [14, 15], which is consistent with the results of the present study. Two potential reasons for the disappointingly poor correlation, even between dimensions with similar contents, might be the different valuing processes (standard gamble versus time-trade-off) and the different numbers of possible health states, with a larger descriptive system in the SF-6D compared with the EQ-5D.

The EQ-5D provides only three levels in each item, giving 243 possible health states, whereas there are between four and six levels per item and 18,000 possible health states in the SF-6D. Therefore, the difference between levels 1 and 2 in the EQ-5D is relatively larger than the difference between levels 1 and 2 in the SF-6D. The smaller difference between health levels in the SF-6D is the main reason for the broader distribution of its dimensional scores compared with the EQ-5D, as observed in both the present study and Brazier's cohorts [10, 14]. This effect might be less important using the new 5-level version of the EQ-5D. Furthermore, the SF-6D instrument includes a broader assessment of HRQoL by accounting for the social functioning dimension, which is not represented in the EQ-5D. The ceiling effect of EQ-5D, which has been demonstrated in previous studies [14, 15, 17, 18], was also observed in the present study. A total of 321 patients with optimal utility scores from the EQ-5D scored lower on the SF-6D. Thus, the larger descriptive system of the SF-6D exhibits higher sensitivity than the EQ-5D in the detection of utility differences in patients with relatively good HRQoL. Furthermore, the ceiling effect of the EQ-5D may decrease with the new 5-level version.

We could not detect differences between the mean and median index scores of the EQ-5D and SF-6D in the entire cohort. Spearman's rho correlation coefficient indicated a moderate correlation between the index scores of the two instruments. However, both the Bland-Altman plot, with many values beyond the limits of agreement, and the ICC indicated poor agreement between the two instruments. Additionally, the plot indicated a ceiling effect for the EQ-5D and a floor effect for the SF-6D, confirming the results from previous studies of different disease groups [14, 15, 18]. Discrepancies in the descriptive systems, the health states, or a combination of both have been mentioned as reasons for the poor agreement between the instruments [17]. The larger descriptive system of the SF-6D allows for more precise descriptions of health states than are possible with the EQ-5D and its ceiling effect. However, the relatively high lower limit of possible scores in the SF-6D results in a floor effect, which decreases its ability to discriminate among serious health states. Additionally, in contrast to SF-6D, the EQ-5D provides the so-called “N3” term, which accounts for the lowest possible health state in any dimension and thus further reduces the EQ-5D index values [10]. Furthermore, the different valuation methods, that is, standard gamble (SF-6D) versus time-trade-off (EQ-5D), may have influenced the agreement between the instruments. Both valuation methods are preference-based and provide a choice between two different outcomes. Only the standard gamble, however, provides a dimension of uncertainty with a risk of death for one of the possible outcomes. Although the standard gamble technique generally valuates health states higher than the time-trade-off method [17], several studies similar to the present study obtained higher scores with the EQ-5D than with the SF-6D, particularly in individuals with mild diseases [35]. In addition to the obvious floor and ceiling effects, peoples' risk attitudes may have also contributed to the observed differences. In general, people are risk-averse for large gains and risk-seeking for small gains, meaning that patients in good health states may obtain lower scores with the standard gamble method compared to the time-trade-off method and vice versa for patients in poor health states [9].

The abilities of both instruments to detect differences in utilities that depend on self-reported disease activity (current symptoms) were rather good in terms of statistical significance and clinical relevance according to Norman's criteria of clinical relevance [33]. This finding confirms the results of previous studies [14, 15, 18]. However, due to the skewed data of 321 patients with an EQ-5D score of 1.0, which reflects a ceiling effect, the median EQ-5D index score in patients without current IBD symptoms was considerably higher than the mean value. Unlike the SF-6D, the EQ-5D was unable to detect utility differences in those patients. Furthermore, in our study, the EQ-5D and its ceiling effect displayed a lower ability than the SF-6D to detect statistically significant utility differences in patient-reported general health in cases in which the patient's health was defined as excellent or very good. A study conducted by Lillegraven et al. was unable to detect this phenomenon in patients with rheumatoid arthritis [18]. Although the AUC for a cut-off between excellent health and very good health was larger when using SF-6D than EQ-5D, the clinical relevance of a utility difference in self-perceived excellent health versus very good health remains unclear due to the overlapping confidence intervals. This overlap might be because the sample size of patients with excellent health in our cohort was too small (*n* = 56) according to our sample size estimation. The ROC analysis revealed no difference between the sensitivities of both instruments to detect differences in utility in poorer health states.

The ability of both MAU instruments to detect a utility difference dependent on the presence or absence of symptoms was also good when the patient groups were stratified by countries. However, the ability to detect utility differences dependent on the presence or absence of flares in the previous year was rather poor, when the data were stratified by countries, a fact that might be in consequence of a type 2 error.

Previous results in the present study and in other population-based IBD cohorts [23, 36] have observed that IBD patients have, on average, a good HRQoL. The ceiling effect of EQ-5D and its poorer discriminatory ability for patients in good health may favour the use of the SF-6D when exploring the utility of unselected IBD patient groups. In contrast, due to the SF-6D's floor effect, the EQ-5D may be more suitable for cohorts of patients selected with severe IBD.

In the present study, the mean differences between the utility index scores for patients with and without disease activity were both considerably higher when using the EQ-5D than the SF-6D instrument. Assuming that these differences are also likely to occur in an IBD patient group before and after interventions, this finding may have implications for the choice of utility instruments in cost-effectiveness analyses. Johnsen et al. showed that, after intervention, the index scores of both the EQ-5D and SF-6D instruments increased in patients with chronic low back pain and degenerative disc disease [16]. The EQ-5D index scores increased twice as much as the SF-6D scores. Relating these effects to costs, calculations performed using the EQ-5D would result in lower costs per QALY than the SF-6D. As a consequence, cost-utility estimates of different interventions in disease cases might yield different results depending on the utility instrument used, as shown in a study by Sach et al. [19]. Thus, the results of cost-effectiveness analyses partly depend on the utility instrument used and must be interpreted with care. Therefore, as recommended by others [13], we agree that utility analyses should always be performed with at least two different utility instruments in IBD patients.

To the best of our knowledge, the present study is the first to compare utility measures between two widely used utility instruments in an unselected IBD patient cohort. The standardised definitions for IBD and relapse and the standardised symptom scores represent the major strengths of this study. The study design, which involved the independent inclusion of patients in each centre, implies that the exclusion of patients from centres with low response rates did not bias the remaining centres, which were regarded as representative of their region. The response rates from the remaining centres were similar to those in other incidence studies [24, 25]. An important limitation is that the registration of utility was performed only once, which made it impossible to evaluate the reliability and responsiveness of the methods in IBD patients. Furthermore, the sample size of the patient group with self-reported excellent health was too small to provide a reliable significance level regarding possible differences between these patients and those with very good health. Lastly, the study did not provide data regarding disease-specific HRQoL, which would be valuable for comparison of the utility instruments.

## 5. Conclusion

In conclusion, the findings of the present study demonstrate poor agreement between the EQ-5D and the SF-6D in patients with IBD. This study confirms the published results from other patient groups. Both instruments provide good sensitivities for detecting utility differences that depend on differences in disease activity, but the instruments cannot be used interchangeably. Furthermore, the SF-6D might be more sensitive at detecting small utility differences in groups of IBD patients with low disease activity. Utility analyses should always be conducted with more than one utility instrument, and cost-utility analyses performed with only one utility instrument must be interpreted with caution.

## Figures and Tables

**Table 1:** Correlations between dimension scores in the SF-6D and EQ-5D.

SF-6D	EQ-5D				
	M	SC	UA	PD	AD
PF	**0.53**	0.34	**0.60**	0.49	0.25
RL	0.47	0.29	**0.64**	0.48	0.38
SF	0.34	0.27	**0.43**	0.41	0.37
P	0.44	0.24	0.52	**0.66**	0.34
MH	0.19	0.12	0.27	0.18	**0.46**
VT	0.27	0.10^*∗*^	0.41	0.41	0.30

Spearman's rho correlation coefficients between dimension scores. Similar dimensions are indicated in bold.

EQ-5D dimensions: M: mobility, SC: self-care, UA: usual activities, PD: pain/discomfort, and AD: anxiety/depression. SF-6D: PF: physical functioning, RL: role limitation, SF: social functioning, P: pain, MH: mental health, and VT: vitality.

**Table 2:** Distribution of health state levels in SF6D and EQ5D (in percentages).

Level	SF-6D							EQ-5D				
	PF	RL	SF	P	MH	VT		MO	SF	UA	PD	AD
1	50.7	67.5	56.7	42.5	25.4	11.6		83.5	95.8	77.6	56.3	67.2
2	26.9	10.3	20.2	21.1	34.5	36.0		16.3	3.8	20.7	38.9	28.9
3	13.9	9.1	12.6	19.9	23.9	30.9		0.3	0.4	1.7	4.8	3.9
4	2.5	13.1	5.7	8.7	12.4	13.4						
5	5.3		4.8	5.9	3.9	8.1						
6	0.7			2								

SF-6D: PF: physical functioning, RL: role limitation, SF: social functioning, P: pain, MH: mental health, and VT: vitality.

EQ-5D: MO: mobility, SF: self-care, UA: usual activities, PD: pain/discomfort, and AD: anxiety/depression.

**Table 3:** Ceiling effects of the EQ-5D. Distribution (%) of the SF-6D health states in 321 patients with EQ-5D index score 1.

Level	PF	RL	SF	P	MH	VT
1	76.0	91.6	81.9	73.8	38.9	19.6
2	19.9	3.7	9.0	17.4	39.6	48.6
3	4.0	3.4	2.2	7.5	13.1	24.9
4		1.2	0.6	0.6	3.7	5.6
5			6.2	0.6	4.7	1.2
6						

PF: physical functioning, RL: role limitation, SF: social functioning, P: pain, MH: mental health, and VT: vitality.

**Table 4:** EQ-5D and SF-6D index scores and subjectively perceived disease activities.

	Current symptoms	Yes	No	Flares in the previous year	Yes	No
EQ-5D scores	Mean (95% CI)	0.69^*∗*^ (0.66–0.72)	0.88 (0.86–0.89)		0.75^*∗*^ (0.71–0.76)	0.82
	Median	0.76	1.0		0.80	0.85

SF-6D scores	Mean (95% CI)	0.71^*∗*^ (0.69–0.72)	0.81 (0.8–0.82)		0.74^*∗*^ (0.71–0.76)	0.78 (0.77–0.79)
	Median	0.70	0.85		0.75	0.80

AUC (95% CI)						
EQ-5D	0.76 (0.72–0.79)			0.61 (0.56–0.66)		
SF-6D	0.72 (0.68–0.76)			0.6 (0.54–0.65)		

Mean (95% confidence interval) and median utility indices with and without symptoms/flares in the previous year; significance of the differences between symptoms/no symptoms and flares/no flares: ^*∗*^ *P* < 0.001 (*t*-test and Mann-Whitney test).

AUC: area under the ROC curve dependent on the information regarding current symptoms or flares in the previous year (95% confidence interval).

**Table 5:** EQ-5D and SF-6D index scores and subjectively perceived disease activities stratified by countries.

		Current symptoms		Flares in the previous year	
		Yes	No	Yes	No
EQ5D scores Mean (95% CI)	Italy	0.76 (0.69–0.84) *N* = 26	0.9 (0.87–0.94)^*∗∗∗*^ *N* = 70	0.78 (0.70–0.86) *N* = 24	0.89 (0.86–0.93)^*∗∗*^ *N* = 72
	Greece	0.53 (0.25–0.82) *N* = 7	0.83 (0.77–0.88)^*∗∗*^ *N* = 67	0.57 (0.16–0.98)^*∗*^ *N* = 7	0.81 (0.75–0.87) *N* = 70
	Israel	0.64 (0.50–0.77) *N* = 17	0.88 (0.76–0.99)^*∗∗*^ *N* = 24	0.73 (0.37–1) *N* = 6	0.79 (0.69–0.88) *N* = 35
	Denmark	0.67 (0.58–0.76) *N* = 26	0.89 (0.84–0.95)^*∗∗∗*^ *N* = 77	0.75 (0.64–0.87) *N* = 37	0.83 (0.78–0.89) *N* = 66
	Norway	0.66 (0.59–0.72) *N* = 99	0.88 (0.85–0.90)^*∗∗∗*^ *N* = 127	0.70 (0.63–0.79) *N* = 53	0.80 (0.76–0.84)^*∗*^ *N* = 173
	Netherlands	0.69 (0.64–075) *N* = 18	0.85 (0.81–090)^*∗∗∗*^ *N* = 138	0.74 (0.62–0.85) *N* = 66	0.79 (0.76–0.83) *N* = 90
	Spain	0.83 (0.76–0.90) *N* = 16	0.92 (0.86–0.97)^*∗*^ *N* = 57	0.89 (0.82–0.95) *N* = 28	0.88 (0.83–0.94) *N* = 45

SF6D scores Mean (95% CI)	Italy	0.69 (0.64–0.75)	0.83 (0.81–0.85)^*∗∗∗*^	0.78 (0.70–0.86)	0.81 (0.79–0.84)^*∗∗*^
	Greece	0.62 (0.46–0.77)	0.80 (0.77–0.84)^*∗∗*^	0.67 (0.36–0.97)	0.79 (0.76–0.83)
	Israel	0.61 (0.56–0.65)	0.64 (0.61–0.67)	0.57 (0.49–0.65)	0.63 (0.61–0.66)^*∗*^
	Denmark	0.74 (0.70–0.79)	0.84 (0.81–0.87)^*∗∗*^	0.77 (0.72–0.82)	0.81 (0.78–0.84)
	Norway	0.71 (0.68–0.74)	0.82 (0.80–0.84)^*∗∗∗*^	0.73 (0.69–0.77)	0.79 (0.77–0.81)^*∗*^
	Netherlands	0.70 (0.69–0.73)	0.80 (0.77–0.83)^*∗∗∗*^	0.71 (0.64–0.79)	0.76 (0.74–0.79)
	Spain	0.75 (0.71–0.79)	0.84 (0.86–0.97)^*∗∗*^	0.79 (0.74–0.85)	0.81 (0.78–0.84)

Mean (95% confidence interval) utility indices with and without symptoms/flares in the previous year; significance of the differences between symptoms/no symptoms and flares/no flares: ^*∗∗∗*^ *P* < 0.001, ^*∗∗*^ *P* < 0.01, and ^*∗*^ *P* < 0.05 (*t*-test and Mann-Whitney test).

**Table 6:** EQ-5D and SF-6D index scores that depend on subjectively perceived health derived from question one in SF-36.

	Self-perceived health (*N*)	Mean	95% CI	Median
EQ-5D	Excellent (56)	0.96	0.92–0.99	1.0
	Very good (212)	0.93^*∗*^	0.91–0.95	1.0
	Good (315)	0.84^*∗∗*^	0.82–0.86	0.85
	Fair (155)	0.62^*∗∗*^	0.58–0.66	0.69
	Poor (31)	0.31^*∗∗*^	0.21–0.41	0.19

SF-6D	Excellent	0.91	0.89–0.94	0.93
	Very good	0.85^*∗∗*^	0.83–0.86	0.89
	Good	0.78^*∗∗*^	0.77–0.80	0.80
	Fair	0.64^*∗∗*^	0.63–0.66	0.62
	Poor	0.54^*∗∗*^	0.52–0.57	0.54

Significance levels of differences per utility instrument between the utility index scores due to different health states: ^*∗*^ *P* = 0.91 (between excellent and very good in EQ-5D); ^*∗∗*^ *P* < 0.001 (ANOVA). Question one in SF-36: “In general, would you say your health is: excellent, very good, good, fair, poor?”
